# Vitamin C Enhances Antiviral Functions of Lung Epithelial Cells

**DOI:** 10.3390/biom11081148

**Published:** 2021-08-03

**Authors:** Trevor Teafatiller, Sudhanshu Agrawal, Gabriela De Robles, Farah Rahmatpanah, Veedamali S. Subramanian, Anshu Agrawal

**Affiliations:** 1 Division of Gastroenterology, Department of Medicine, University of California, Irvine, CA 92697, USA; tteafati@uci.edu; 2 Division of Basic and Clinical Immunology, Department of Medicine, University of California, Irvine, CA 92697, USA; sagrawal@uci.edu; 3 Department of Pathology, University of California, Irvine, CA 92697, USA; gderoble@uci.edu (G.D.R.); frahmatp@uci.edu (F.R.)

**Keywords:** vitamin C, airway epithelial cells, antiviral responses, ISGs, vitamin C transporters, *GULO*-KO mice

## Abstract

Vitamin C is well documented to have antiviral functions; however, there is limited information about its effect on airway epithelial cells—the first cells to encounter infections. Here, we examined the effect of vitamin C on human bronchial epithelium transformed with Ad12-SV40 2B (BEAS-2B) cells, and observed that sodium-dependent vitamin C transporter 2 (SVCT2) was the primary vitamin C transporter. Transcriptomic analysis revealed that treating BEAS-2B cells with vitamin C led to a significant upregulation of several metabolic pathways and interferon-stimulated genes (ISGs) along with a downregulation of pathways involved in lung injury and inflammation. Remarkably, vitamin C also enhanced the expression of the viral-sensing receptors retinoic acid-inducible gene 1 (RIG-1) and melanoma differentiation-associated protein 5 (MDA-5), which was confirmed at the protein and functional levels. In addition, the lungs of l-gulono-γ-lactone oxidase knockout (*GULO*-KO) mice also displayed a marked decrease in these genes compared to wild-type controls. Collectively, our findings indicate that vitamin C acts at multiple levels to exert its antiviral and protective functions in the lungs.

## 1. Introduction

Vitamin C is an essential nutrient that plays an important role in metabolic, redox, and epigenetic pathways. Human cells cannot produce vitamin C endogenously due to mutations in the gene for l-gulono-γ-lactone oxidase (GULO) enzyme synthesis, and thus, they obtain the vitamin from their surroundings following intestinal absorption and blood circulation via sodium-dependent vitamin C transporters (SVCT1 and SVCT2) [1,2,3,4,5]. The antioxidant properties of vitamin C are considered highly beneficial for chronic lung diseases such as chronic obstructive pulmonary disease (COPD), helping to reduce lung damage. The beneficial effects of vitamin C in the treatment and prevention of respiratory viral infections such as the common cold, influenza, etc., are also well documented [6,7,8]. In addition, growing evidence suggests that vitamin C may play an adjunctive role in the treatment of the recent COVID-19 pandemic [9,10]. Studies using murine models have shown that vitamin C can modulate the production of the antiviral cytokine interferon alpha (IFN-α), and protect the mice from influenza-induced lung injury [8]. Most of the beneficial effects of vitamin C on viral infections are attributed to enhanced immune responses [11], and there is limited knowledge about the effects of vitamin C on the functions of lung epithelial cells, the first cells to encounter the pathogens. Furthermore, the respiratory epithelium not only lines the trachea, bronchus, and bronchioles, serving as a barrier to the outside environment, but is also the site of replication of most respiratory viruses and bacteria. In addition to the barrier function, the epithelium senses and responds to viral infections by upregulating various antiviral molecules, such as MHC-I (major histocompatibility complex class I), type I interferons, etc. [12]. The molecules secreted by these cells modulate the response of immune cells such as dendritic cells and macrophages that are present in the vicinity [13,14]. Therefore, our aim in this study is to investigate the effect of vitamin C on the functions of airway epithelial cells, with a particular focus on antiviral responses. This was investigated using in vitro (normal human bronchial epithelial BEAS-2B cells) and in vivo (*GULO*-KO mice lacking the GULO enzyme similar to humans) as model systems.

## 2. Materials and Methods

### 2.1. Materials

The SV40 large T-antigen-immortalized, human-derived lung bronchial epithelial cell line BEAS-2B was obtained from the American Type Culture Collection (ATCC, Rockville, MD, USA). Primary human bronchial epithelial cells were obtained from Lonza (Walkersville, MD, USA). ^14^C-AA (specific activity of 2.8 mCi/mmol; radiochemical purity > 97%) was from PerkinElmer (Boston, MA, USA). DNA oligonucleotide primers used in this study were synthesized by Integrated DNA Technologies (San Diego, CA, USA). All biochemical and molecular biology reagents were from commercial sources.

### 2.2. Cell Culture, Stimulation and Uptake Assay

The BEAS-2B and primary cells were cultured in BEGM (bronchial epithelial cell growth medium) (Lonza) and used between 2 and 10 passages. Cells were treated with vitamin C (100 μM) for 24 h and used for uptake and molecular biological investigations. ^14^C-AA uptake (30 min) was measured in BEAS-2B cells at 37 °C, as described previously [15,16]. Twenty-four hours after the addition of vitamin C, cells were stimulated with either poly I:C (high molecular weight)/LyoVec (InvivoGen, San Diego, CA, USA) at 1 μg/mL or IFN-α (interferon source) at 100 units/Ml. Cells were used for qPCR, and the supernatants collected were assayed for IL-6 and IL-8 using specific ELISA (enzyme-linked immunosorbent assay; BioLegend, San Diego, CA, USA).

### 2.3. RNA Sequencing and Analysis

BEAS-2B cells were exposed to vitamin C (100 μM) for 24 h. Subsequently, the RNA collected was sent to MedGenome (San Diego, CA, USA) for library preparation and RNA sequencing. Sequencing reads were aligned to genome reference files from Ensembl using the Strand NGS 3.1 (http://www.strand-ngs.com/) sequencing analysis package. Gene expression was quantified from deduplicated sequencing reads for each sample. Pooled analysis was performed using the Audic–Claverie (AC) test, which pools together raw counts across three sets of vitamin-C-treated and untreated BEAS-2B cells, separately, and tests the differences between them based on a Poisson assumption of the distribution of counts. A multiple testing correction, Benjamini–Hochberg FDR (false discovery rate) of 0.05, and a maximum *p*-value cutoff of 0.05 were used as described previously [14,17]. The genes of the AC test were ranked based on adjusted *p*-value (FDR corrected), and cutoff was set at *p* adj < 0.05 to select for statistically significant differentially expressed genes between the vitamin-C-treated and untreated BEAS-2B cells. Pathway and network analysis was carried out using Ingenuity Pathway Analysis software (Qiagen, Germantown, MD, USA). The interferon-stimulated genes were determined using the Interferome database version 2 (http://www.interferome.org/interferome/home.jspx) [18,19].

### 2.4. GULO-KO Mice

We used the *GULO*-KO mice (which cannot produce vitamin C endogenously due to lack of l-gulono-γ-lactone oxidase (GULO) enzyme) and their littermate control mice in this study [20]. *GULO*-KO mice were maintained by supplementing vitamin C (0.33 g/L) in their drinking water. Vitamin C supplementation was stopped for 1 week prior to euthanasia and harvesting of the lungs. The animal protocol used in this study was approved by the Institutional Animal Care and Use Committee (IACUC), University of California, Irvine, CA, USA.

### 2.5. RT-qPCR Analysis

Two micrograms of total RNA isolated from human primary lung epithelial cells or BEAS-2B cells or from murine lungs were treated with DNase I (Invitrogen) followed by cDNA synthesis using the iScript cDNA synthesis kit (Bio-Rad, Hercules, CA, USA). The gene primers used are shown in Table 1. RT-qPCR conditions and quantification analysis were performed as described previously [15,16].

### 2.6. Flow Cytometry

Vitamin-C-treated and untreated BEAS-2B cells were stained intracellularly for MX1 and RIG-1 (Biorbyt, St. Louis, MO, USA), and MDA-5 (Thermo Fisher, Waltham, MA, USA) using specific antibodies. Appropriate isotype controls were used for all antibodies. Analysis was performed using FlowJo software Version 10.8 (Ashland, OR, USA).

### 2.7. Western Blot Analysis

Cell lysates were prepared from vitamin-C-treated and untreated BEAS-2B cells. Sixty micrograms of protein samples were separated in NuPAGE 4–12% Bis-Tris gradient minigels (Invitrogen, Carlsbad, CA, USA), then transferred onto Immobilon polyvinylidene difluoride membrane (Fisher Scientific, Chino, CA, USA). The well-characterized anti-SVCT-2 (1:200 dilutions) and β-actin (1:2000 dilutions) antibodies were used as primary antibodies. The anti-rabbit IRDye-800 and anti-mouse IRDye-680 (both at 1:30,000 dilutions) were used as secondary antibodies. The immunoreactive bands were quantified using the Odyssey infrared imaging system (LI-COR Biosciences, Lincoln, NE, USA).

### 2.8. Statistical Analysis

^14^C-AA uptake data represent the results of at least three separate experiments with multiple determinations and are expressed in percentage as means ± SE. Western blot, flow cytometry, and RT-qPCR analysis were performed using at least three or more separate sample preparations. Data were analyzed using Student’s *t*-test, with statistical significance set at *p* < 0.05.

## 3. Results

### 3.1. SVCT2 Is the Predominant Vitamin C Transporter in the Lungs

**Figure 1:**
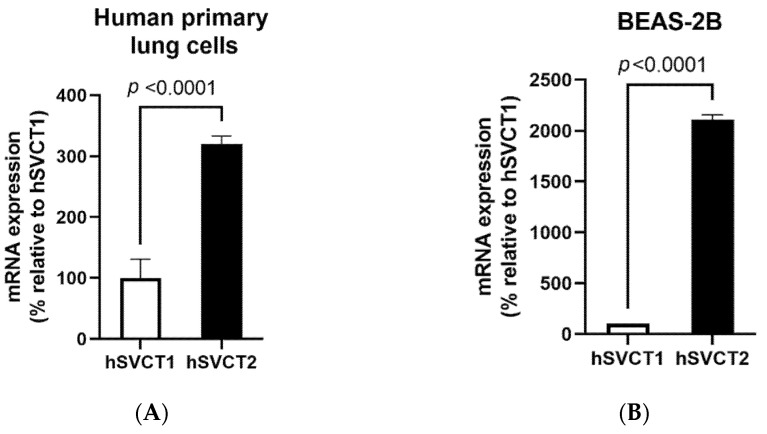

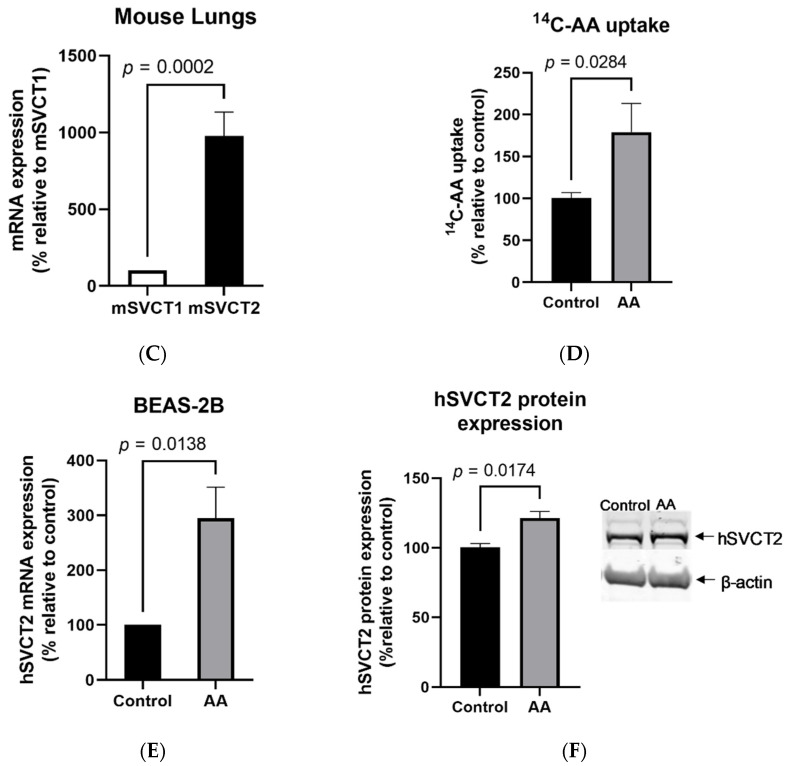
Vitamin C transporter expression and function in the lungs. The expression of the vitamin C transporters SVCT1 and SVCT2 was determined using RT-qPCR in (**A**) human primary bronchial epithelial cells; (**B**) BEAS-2B cells; and (**C**) native wild-type mouse lungs. (**D**) To determine the effect of vitamin C/ascorbic acid (AA) treatment on ^14^C-AA uptake and SVCT2 expression levels, the BEAS-2B cells were exposed to vitamin C (100 μM, 24 h) and carrier-mediated ^14^C-AA uptake was performed. Expression of (**E**) SVCT2 mRNA and (**F**) protein levels were determined for isolated mRNA and proteins by RT-qPCR and Western blot analysis, respectively. Data are means ± SE of at least 3–4 independent sample preparations.

Previous studies have suggested that SVCT2 may be the predominant vitamin C receptor in the lungs [21]. We examined whether this was the case in our studies. The relative expression of vitamin C transporter (SVCT1 and SVCT2) mRNA was examined in the human primary bronchial epithelial cells, BEAS-2B cells, and mouse lungs to determine the specific roles of these transporters in these model systems. The results showed that both SVCT1 and SVCT2 are expressed in human lung cells and mouse lungs, with the expression of SVCT2 being markedly higher than that of SVCT1 (Figure 1A–C). These results clearly show that the SVCT2 system is the predominant vitamin C transporter expressed in the selected human and murine models and, therefore, further analysis was focused on the effect of vitamin C treatment on the functional expression of SVCT2 in BEAS-2B cells.

Subsequently, we confirmed the functional activity of the transporters. For this, we determined the effect of vitamin C treatment on the ^14^C-AA uptake in BEAS-2B cells. Our results showed a significant (*p* < 0.05) increase in AA uptake in vitamin-C-treated compared to untreated control cells (Figure 1D). This upregulation was associated with a marked increase in the levels of expression of SVCT2 mRNA (Figure 1E) and proteins (Figure 1F) in vitamin-C-treated compared to untreated control cells. These findings indicate that the induced AA uptake by vitamin C treatment is mediated via SVCT2 in human lung cells.

### 3.2. Transcriptomic Changes in BEAS-2B Cells after Treatment with Vitamin C

**Figure 2:**
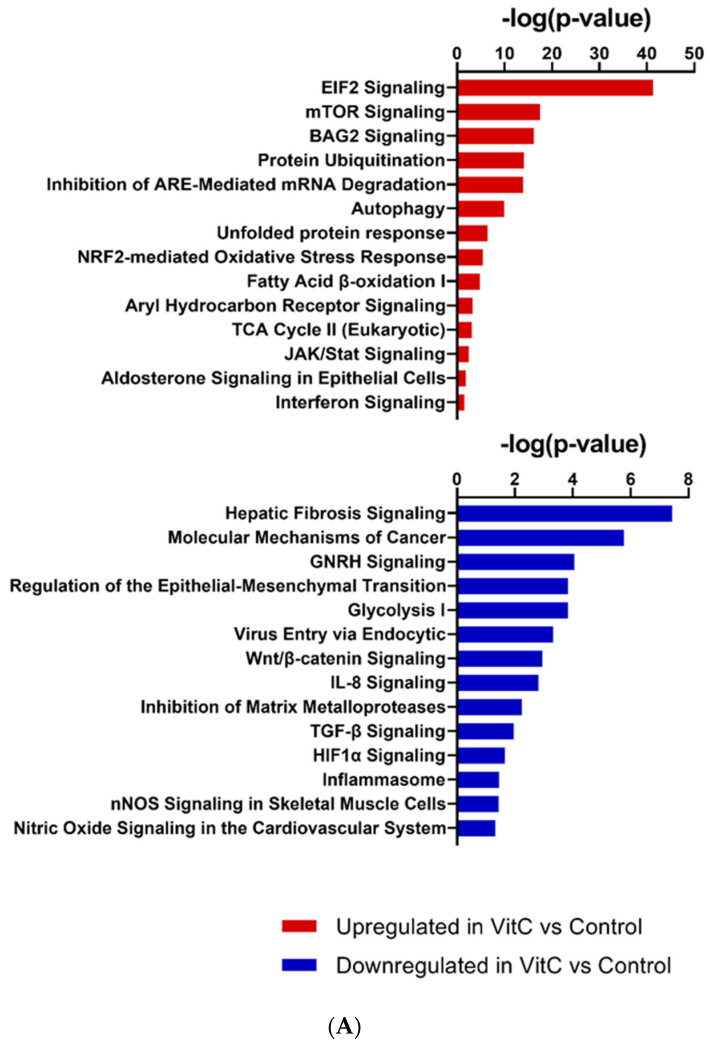

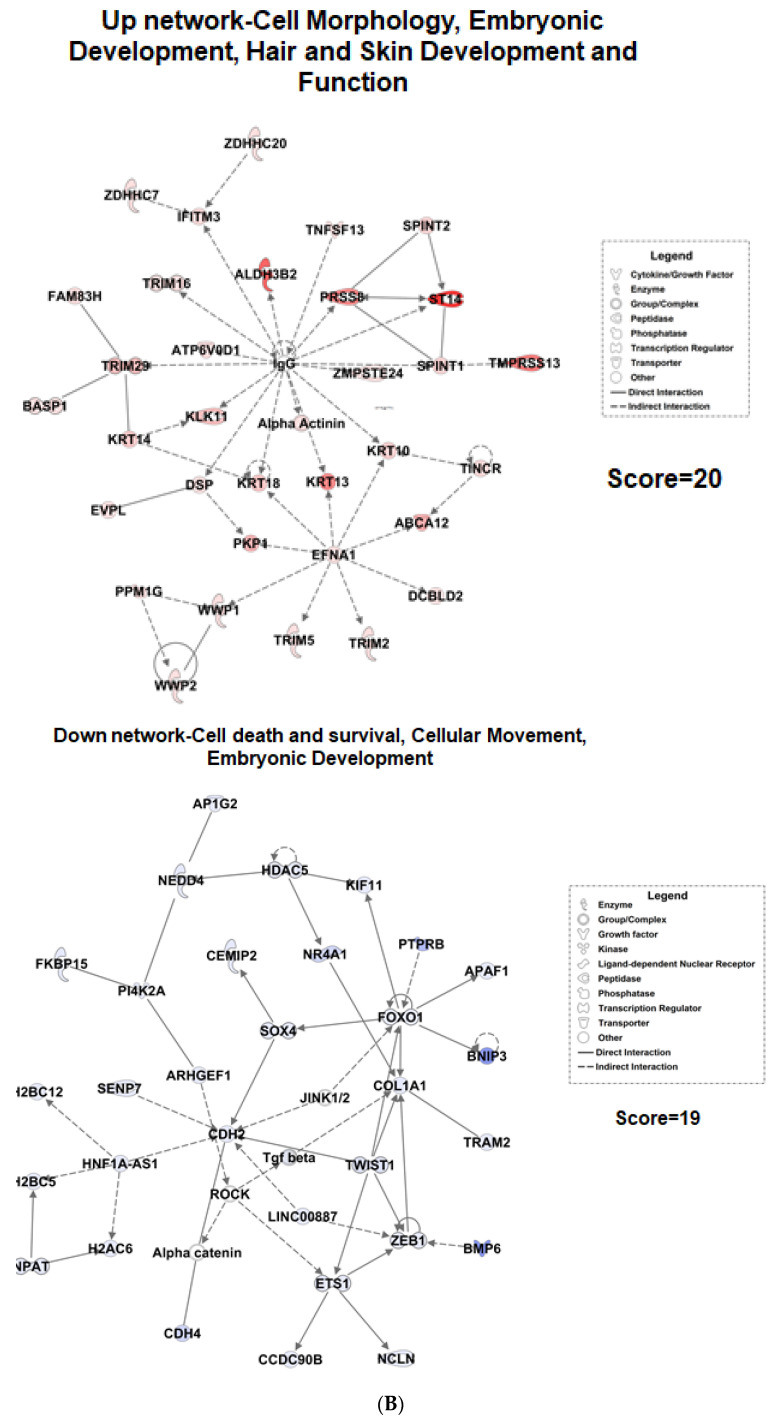

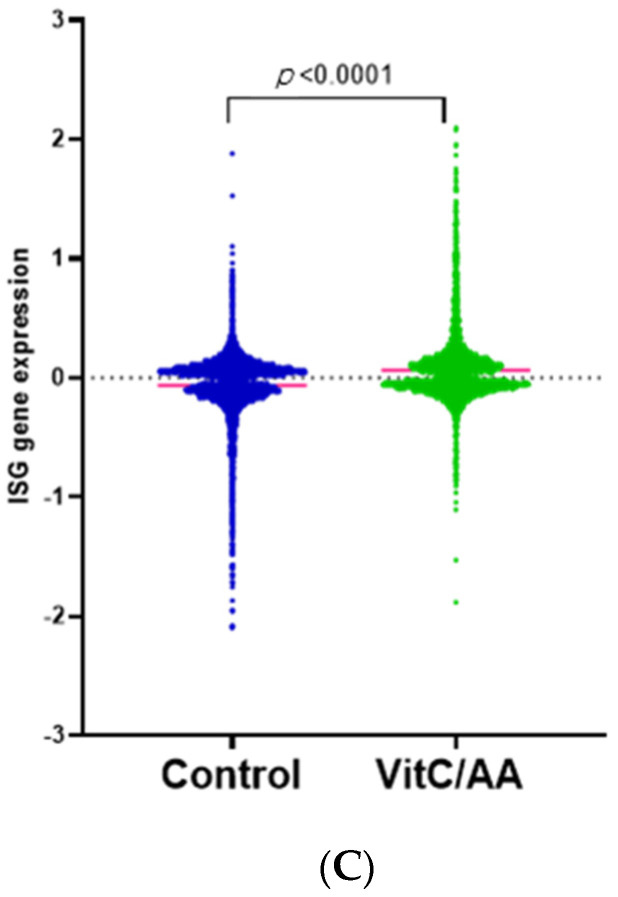
Transcriptomic changes in BEAS-2B cells after treatment with vitamin C. RNA-sequencing was performed on vitamin-C-treated and untreated BEAS-2B cells. (**A**) Selected canonical pathways of upregulated and downregulated differentially transcribed genes in control (*n* = 3) and vitamin-C-treated (*n* = 3) cells after Ingenuity Pathway Analysis (−log10 *p* value ≥ 1.30). (**B**) Gene network analysis of up- and downregulated genes in control (*n* = 3) and vitamin-C-treated (*n* = 3) cells. The networks shown are among those with the highest significance of connections between molecules in the network, as indicated by their score. Blue nodes indicate downregulated and red nodes indicate upregulated gene expression in vitC-treated vs. control cells. Darker shades of the nodes indicate larger differential expression ratios. Dotted lines represent indirect interactions, while solid lines represent direct interactions. (**C**) RNA expression of ISGs as determined by the Interferome database. Based on the Interferome database of more than 25,000 ISGs, 7838 differentially expressed genes were identified in vitC-treated vs. control cells, 4313 of which were ISGs. These ISGs were expressed at statistically lower levels (*t*-test *p*-value < 0.0001) in control cells (*n* = 3) compared to vitC-treated cells (*n* = 3).

RNA sequencing was performed on BEAS-2B cells treated with physiological concentrations of vitamin C for 24 h. The total number of significant genes changed with an adjusted *p*-value less than 0.05 was 7838 (Supplementary Table S1), out of which 3712 were upregulated and 4126 were downregulated. Pathway analysis revealed that major pathways showing upregulation included the tricarboxylic acid (TCA) cycle, fatty acid oxidation, nuclear factor erythroid 2–related factor 2 (NRF2), mammalian target of rapamycin (mTOR), etc., consistent with the role of vitamin C as an antioxidant. In addition, vitamin C prevented mRNA decay, as the inhibition of antioxidant responsive element (ARE)-mediated decay of mRNA was among the top three pathways to be upregulated after vitamin C treatment (Figure 2A). This pathway has been documented to play a major role in regulating inflammation [22,23]. Furthermore, vitamin C upregulated both the BAG-2 and ubiquitination pathways. These two pathways are involved in protecting the cell from damage caused by misfolded proteins [24]. Additionally, BAG-2 is an inhibitor of the Hsp70-binding E3 ubiquitin ligase CHIP (carboxyl-terminus of Hsp70-interacting protein) [25]. Several pathways that play important roles in antiviral responses were also upregulated; these include eIF2 signaling, autophagy, interferon response, and the JAK/STAT pathway. Interestingly, vitamin C also upregulated aldosterone signaling in epithelial cells. Decreased synthesis and expression of aldosterone is associated with increased lung injury [26]. Network analysis also supported the role of vitamin C in lung injury (Figure 2B). One of the molecules upregulated in the network was PRSS8 (protease serine 8)—a membrane-bound channel-activating protease 1 that has been reported to play a crucial role in the removal of fluid from the alveolar space. The PRSS8-interacting *ST14* gene was also upregulated. The xenobiotic response via the aryl hydrocarbon receptor (AHR) pathway was upregulated, and aldehyde dehydrogenase 3B (ALDH3B)—the molecule in the network—is an integral part of this pathway. Several tripartite motif proteins (TRIMs) are also upregulated in the network, and TRIM29 is reported to be involved in macrophage activation in response to respiratory infections [27].

Amongst the downregulated pathways, fibrosis-related pathways such as the fibrosis signaling, TGF-β signaling, Wnt-β catenin, and matrix metalloprotease signaling pathways displayed major changes (Figure 2A). Network analysis also showed the TGF-β network to be downregulated (Figure 2B). The downregulated network also had genes that prevent epithelial–mesenchymal transition, such as the TWIST1 (Twist family BHLH transcription factor 1) [28]. Pathway analysis supported this observation, since cancer-related pathways such as GnRH and epithelial–mesenchymal transition were downregulated after treatment with vitamin C. Additionally, the inflammasome, nitric oxide, and viral entry pathways were also downregulated.

To investigate the role of vitamin C in antiviral responses, we aimed to determine whether there were changes in interferon-stimulated genes (ISGs) after vitamin C treatment, using the Interferome database [18,19]. ISGs are thought to be induced by type I interferons and play a significant role in inhibiting viral replication [27]. Recent advances indicate that the ISGs can be expressed at the basal level, and may be upregulated independently of interferon signaling. Our results show that treatment with vitamin C leads to significant upregulation (*p* < 0.0001) in ISGs (Figure 2C, Supplementary Table S2). Several interferon-induced proteins with tetratricopeptide repeats (IFIT) that inhibit viral translation—such as IFIT1, 2, 3, and 5—were upregulated upon treatment with vitamin C. Other viral restriction factors (interferon-induced transmembrane proteins; IFITMs) were also upregulated. In addition, vitamin C treatment led to the significant upregulation of both interferon-induced helicase 1 (*IFIH1*) and DExD/H-Box helicase 58 (*DDX58*), which are genes for melanoma differentiation-associated protein 5 (MDA-5) and retinoic acid-inducible gene I (RIG-1) receptors, respectively. These pathogen-recognition receptors are present in the cytoplasm and can sense viral nucleic acids [29]. Altogether, these results indicate that vitamin C not only improves lung health and prevents lung injury, but also enhances viral recognition and response in airway epithelial cells.

### 3.3. Validation of RNA sequencing Data in BEAS-2B Cells and GULO-KO Mice

**Figure 3:**
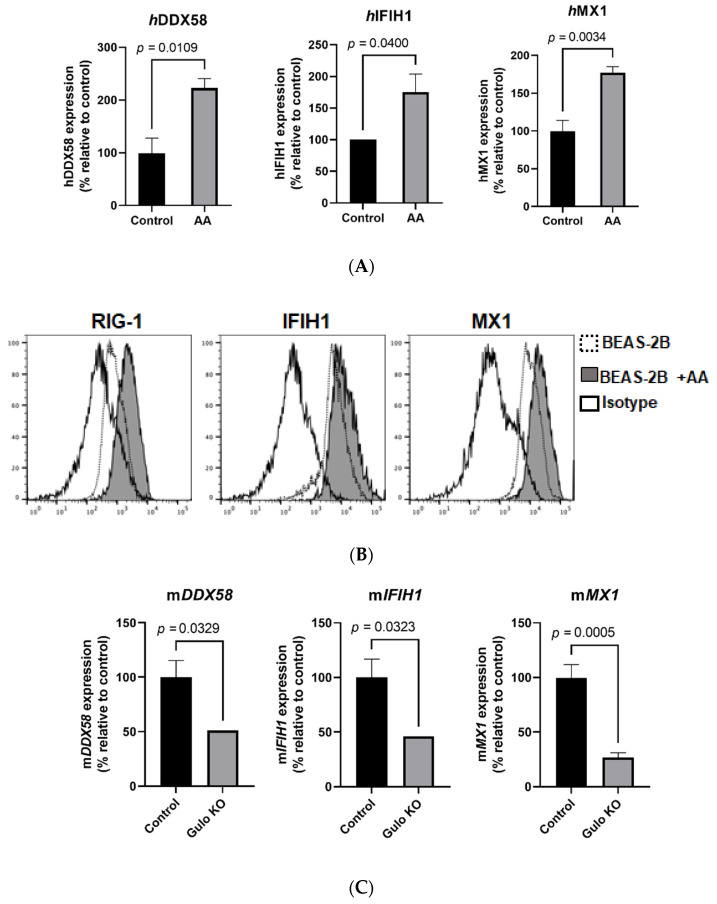
Effect of vitamin C on antiviral gene expression in in vitro and in vivo model systems. RT-qPCR was performed on BEAS-2B cells in the presence and absence of vitamin C/AA. (**A**) Bar graphs depict the expression of RIG-1, IFIH1/MDA-5, and MX1. Data are means ± SE of at least 4 experiments performed on separately isolated samples. (**B**) Histograms depict the expression of these molecules at the protein level using flow cytometry. (**C**) Bar graphs depict the effect of vitamin C deficiency on the expression of murine RIG-1, IFIH1/MDA-5, and MX1 mRNA in the lungs of *GULO*-KO mice, as determined by RT-qPCR. Data are means ± SE of at least 4–5 sets of mice.

We confirmed the upregulation of the viral-sensing receptors RIG-1 and MDA-5, as well as the antiviral gene MX1, via RT-qPCR and flow cytometry in BEAS-2B cells. Our results demonstrate that treatment with vitamin C resulted in a significant upregulation of the human retinoic acid-inducible gene 1 (h*RIG-1*) (*p* < 0.02) and human melanoma differentiation-associated protein 5 (h*MDA-5*) genes (Figure 3A). Similar results were obtained at the protein level using flow cytometry, where vitamin C treatment also led to significant upregulation of RIG-1 and MDA-5 (Figure 3B). In addition to viral-sensing receptors, vitamin C also enhanced the expression of hMX1 (*p* < 0.01) at the mRNA (Figure 3A) and protein levels (Figure 3B). These in vitro responses confirm the vitamin-C-induced changes observed via RNA sequencing.

We next determined the change in these genes in *GULO*-KO mice to confirm our in vitro findings. The *GULO*-KO mice (3–4 months old) were maintained without vitamin C supplementation in the drinking water for 1 week in order to induce vitamin C deficiency. Subsequently, the lungs from these mice, as well as control mice, were used to determine the expression of murine mMX1, mRIG-1, and mMDA-5 mRNA levels. Our results demonstrate that mMX1, mMDA-5, and mRIG-1 mRNA levels were markedly decreased in the lungs of *GULO*-KO mice compared to their control littermates (Figure 3C). These in vivo results confirm the role of vitamin C in the regulation of antiviral gene expression in the lungs.

### 3.4. Vitamin C Treatment Enhances the Response of BEAS-2B Cells to the Antiviral Ligand Poly I:C

**Figure 4:**
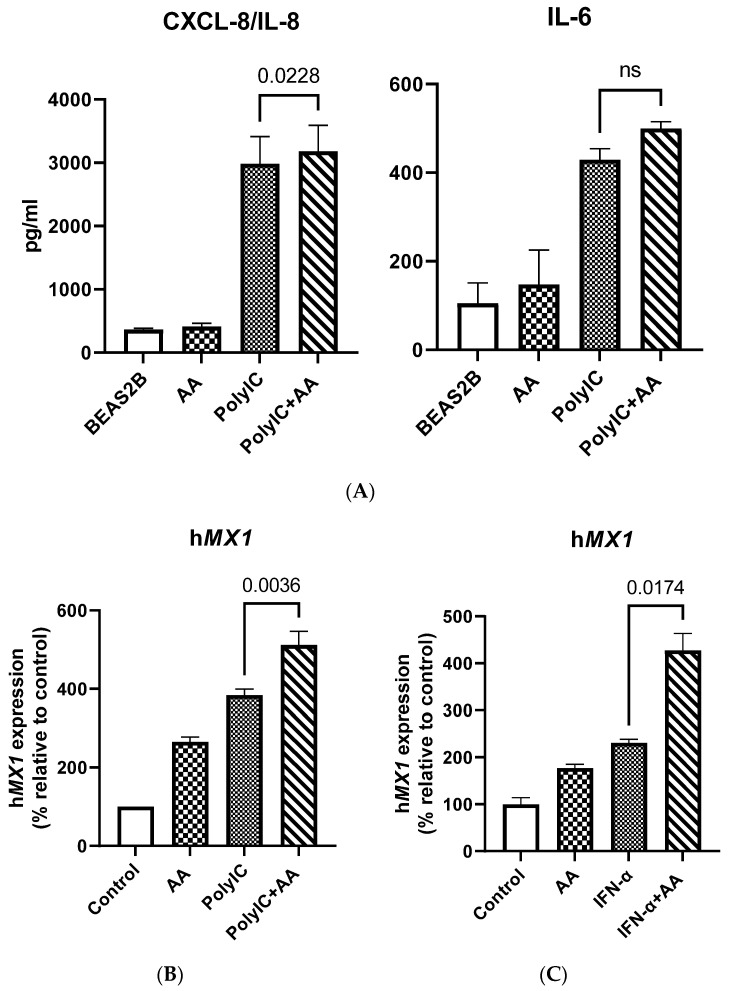
Vitamin C treatment enhances the response of BEAS-2B cells to the antiviral ligand poly I:C. (**A**) Bar graphs depict the levels of CXCL-8/IL-8 and IL-6 as determined by ELISA. (**B**) BEAS-2B cells were stimulated with poly I:C in the presence or absence of vitamin C for 24 h. Bar graph depicts the expression of the MX dynamin like GTPase 1 (MX1) gene. (**C**) BEAS-2B cells were stimulated with IFN-α in the presence or absence of vitamin C for 24 h. Bar graph depicts the expression of the MX1 gene. Data are means ± SE of at least 4 separate experiments.

Since we observed increased expression of RIG-1 and MDA-5, we wanted to confirm whether this led to an enhancement of responses against their ligands. To investigate this, BEAS-2B cells were exposed to vitamin C (100 μM) overnight, and subsequently stimulated with polyinosinic:polycytidylic acid (poly I:C) (1 μg/mL) for 24 h. Supernatants collected were assayed for IL-8 and IL-6 via ELISA. Cells were used for RT-qPCR analysis. Stimulation with poly I:C resulted in increased secretion of both IL-6 and IL-8 (Figure 4A). However, the addition of vitamin C led to a significant increase in IL-8 secretion in response to poly I:C (Figure 4A). IL-6 secretion, though increased, was not significant. We also observed an increase in MX1 mRNA expression due to poly I:C (Figure 4B). Increased ISG response in the form of MX1 was also observed when BEAS-2B cells were stimulated with IFN-α (Figure 4C). In summary, these data indicate that vitamin C enhances the response of airway epithelial cells to viral nucleic acids and type I IFNs.

## 4. Discussion

Vitamin C is well documented to exert a protective effect in the lungs, particularly against viral infections [6,8,11,30]. The antiviral effects of vitamin C were first highlighted by Linus Pauling in 1970, when he demonstrated its benefit in preventing and alleviating the common cold. Even though his conclusions were based on a single clinical trial, and may have been over-optimistic, they paved the way for the investigation of other benefits of vitamin C, beyond the prevention of scurvy. Since then, several studies have shown that vitamin C improves survival in different murine models of lethal infection [6,8]. For example, viral titers were reduced, and mortality was reduced by 50% in mice infected with Venezuelan encephalitis virus and treated with vitamin C (50 mg/kg body weight) [31]. Vitamin C also reduced mortality in stressed mice with H1N1-induced viral pneumonia, in a dose-dependent manner [32]. Furthermore, pathological changes in the lungs were also higher in *GULO*-KO mice infected with influenza [33]. Human trials with vitamin C have reported modest benefits [6]. Despite these studies, there is limited information about the effect of vitamin C on airway epithelial cells. Here, we investigated the effect of the vitamin on AECs, as these cells are important players in antiviral responses. Our results indicate that SVCT2 is the primary vitamin C transporter in the lung epithelial cells. This is consistent with previous studies that also reported SVCT2 to be the major transporter in AECs [34]. Furthermore, we also find that the transporter is functional, suggesting that epithelial cells in the lung can absorb vitamin C from the blood vessels.

Transcriptomic analysis of the effect of vitamin C on AECs indicates that vitamin C enhances energy generation via mitochondrial respiratory pathways and prevents the formation of reactive oxygen species via NRF2 pathways. This is related to vitamin C’s antioxidant function [35]. Vitamin C may thus be effective in reducing virus-induced oxidative lung injury [36]. In an experimental model of RSV, antioxidant administration was shown to reduce inflammation and injury in the lungs [37]. Several other upregulated pathways also support the role of vitamin C as an antiviral agent. The eIF2 pathway was amongst the top three pathways that were upregulated in BEAS-2B cells following vitamin C treatment. It has been shown that eIF2-mediated translation control can be activated via cellular stress, including viral infection [38]. This leads to reduced protein synthesis and, consequently, a reduction in viral replication. Similarly, autophagy is another upregulated pathway that is a powerful antiviral tool; it not only helps in the degradation of viral cargo, but also activates the innate immune responses that result in the production of type I interferons [39]. The interferon response pathway, as well as the JAK/STAT pathway that is downstream of interferon signaling, were both upregulated (Figure 2A). We have confirmed this at the functional level since the addition of vitamin C enhanced the expression of MX dynamin like GTPase 1 (MX1) in response to type I interferon (Figure 4C). Remarkably, vitamin C upregulated the expression of the viral-sensing receptors RIG-1 and MDA-5 in both humans and mice (Figure 3A–C). Furthermore, vitamin C also led to the upregulation of several other ISGs, including OAS1, 2, and 3, which sense RNA and lead to the synthesis of RNase, which can degrade viral RNA [27,40]. Furthermore, IFITMs that have been reported to be highly expressed in barrier cells such as epithelial cells were also upregulated. These proteins prevent the viruses from entering the cells by restricting their transport across the lipid bilayer [41]. The activity of TLRs is also enhanced by vitamin C treatment, as MYD88 (the adaptor molecule downstream of TLRs) was also upregulated. Apart from these well-known ISGs, several others were also upregulated whose modes of action are not known. In addition, many ISGs can synergize together to enhance the antiviral functions.

Vitamin C also modulated several pathways that protect the lungs from cancer, injury, and fibrosis. Primary amongst these was the Wnt/β-catenin pathway, which was downregulated by vitamin C. Cumulative studies point to an involvement of the Wnt/β-catenin pathway in chronic lung pathologies such as idiopathic pulmonary fibrosis, asthma, chronic obstructive pulmonary disease (COPD), etc. In pulmonary fibrosis, the activation of the Wnt/β-catenin pathway results in the production of proinflammatory cytokines by the airway epithelial cells [42]. In asthma, it is involved in early development and airway remodeling [43]. In contrast to the Wnt/β-catenin pathwaty, the aldosterone pathway was upregulated. Aldosterone prevents alveolar edema by regulating epithelial sodium channels and, thus, preventing lung injury [26]. Pathways regulating cancer development, such as GnRH and epithelial–mesenchymal transition, were downregulated. The GnRH pathway has been shown to play a role in lung cancer, where aldosterone helps in the maintenance of cancer stem cells by upregulating the s c-Jun N-terminal kinase (JNK) signaling pathway [44]. Pathways inducing inflammation—such as inflammasomes and nitric oxide synthesis—were all inhibited by vitamin C.

The recent COVID-19 pandemic has once again thrown the spotlight on vitamin C as an antiviral agent. Previous studies have reported that high-dose vitamin C was effective in shortening the stay of patients in intensive care units (ICUs) and reducing the mortality of patients with sepsis, severe influenza, and acute lung injury [45,46,47]. Since severe SARS-CoV-2 infection also causes inflammation like sepsis, as well as acute respiratory distress syndrome (ARDS), several studies have tried using high doses of vitamin C to help ameliorate these symptoms. Initial trials were in China, and beneficial effects of high-dose intravenous (IV) vitamin C were reported. Since then, several clinical trials have been started. Most of them have not reported a major difference in mortality, duration of mechanical ventilation, or organ failure between those treated with vitamin C and controls [10,48]. Improvements in oxygenation have been reported [10]. Most of these trials had a low number of patients, and the baseline vitamin C concentrations were not investigated. Furthermore, the administration of vitamin C was only initiated in the later stages of the disease when the patients were critically ill. At that stage, only the anti-inflammatory effects of vitamin C will be useful. The early beneficial effects of vitamin C have not been investigated. Our results indicate that vitamin C may work both as a preventative and—in the early stages of COVID-19 infection—by enhancing viral recognition and response.

## 5. Conclusions

In summary, our results indicate that vitamin C has a profound effect on the functions of AECs. The vitamin not only enhances their antiviral activity, but also prevents lung fibrosis and injury. Our data also suggest that targeting vitamin C delivery to AECs via inhalation or nebulization may be effective in reducing viral infections. This may especially be useful in the elderly and those suffering from diseases such as diabetes and hypertension, where the transport of the vitamin C via the intestine is compromised [49].

## Figures and Tables

**Table 1:** Combination of primers used to perform RT-qPCR.

Gene	Forward; Reverse Primer Sequence (5′-3′)	Product Size (bp)
Human primers		
hSVCT1	TCATCCTCTTCTCCCAGTACCT; AGAGCAGCCACACGGTCAT	141
hSVCT2	TCTTTGTGCTTGGATTTTCGAT; ACGTTCAACACTTGATCGATTC	106
hMX1	GGTGGTCCCCAGTAATGTGG; CGTCAAGATTCCGATGGTCCT	97
hDDX58	TGTGCTCCTACAGGTTGTGGA; CACTGGGATCTGATTCGCAAAA	120
hIFIH1	TCGAATGGGTATTCCACAGACG; GTGGCGACTGTCCTCTGAA	152
hβ-actin	CATCCTGCGTCTGGACCT; TAATGTCACGCACGATTTCC	116
Mouse primers		
mSVCT1	CAGCAGGGACTTCCACCA; CCACACAGGTGAAGATGGTA	240
mSVCT2	AACGGCAGAGCTGTTGGA; GAAAATCGTCAGCATGGCAA	238
mMX1	GACCATAGGGGTCTTGACCAA; AGACTTGCTCTTTCTGAAAAGCC	182
mDDX58	AAGAGCCAGAGTGTCAGAATCT; AGCTCCAGTTGGTAATTTCTTGG	106
mIFIH1	AGATCAACACCTGTGGTAACACC; CTCTAGGGCCTCCACGAACA	107
mβ-actin	ATCCTCTTCCTCCCTGGA; TTCATGGATGCCACAGGA	136

## Data Availability

The data presented in this study are available within the article or the Supplementary Materials.

## Supplementary Materials

The following are available online at https://www.mdpi.com/article/10.3390/biom11081148/s1: Table S1: Differential gene expression; Table S2: List of ISGs with differential expression.

## References

[1] Daruwala R., Song J., Koh W.S., Rumsey S.C., Levine M. (1999). Cloning and functional characterization of the human sodium-dependent vitamin C transporters hSVCT1 and hSVCT2. FEBS Lett..

[2] Rajan D.P., Huang W., Dutta B., Devoe L.D., Leibach F.H., Ganapathy V., Prasad P.D. (1999). Human placental sodium-dependent vitamin C transporter (SVCT2): Molecular cloning and transport function. Biochem. Biophys. Res. Commun..

[3] Wang Y., Mackenzie B., Tsukaguchi H., Weremowicz S., Morton C.C., Hediger M.A. (2000). Human vitamin C (L-ascorbic acid) transporter SVCT1. Biochem. Biophys. Res. Commun..

[4] Maulén N.P., Henríquez E.A., Kempe S., Cárcamo J.G., Schmid-Kotsas A., Bachem M., Grünert A., Bustamante M.E., Nualart F., Vera J.C. (2003). Up-regulation and polarized expression of the sodium-ascorbic acid transporter SVCT1 in post-confluent differentiated CaCo-2 cells. J. Biol. Chem..

[5] Boyer J.C., Campbell C.E., Sigurdson W.J., Kuo S.M. (2005). Polarized localization of vitamin C transporters, SVCT1 and SVCT2, in epithelial cells. Biochem. Biophys. Res. Commun..

[6] Colunga Biancatelli R.M.L., Berrill M., Marik P.E. (2020). The antiviral properties of vitamin C. Expert Rev. Anti Infect. Ther..

[7] Hemila H., Chalker E. (2013). Vitamin C for preventing and treating the common cold. Cochrane Database Syst. Rev..

[8] Kim Y., Kim H., Bae S., Choi J., Lim S.Y., Lee N., Kong J.M., Hwang Y.I., Kang J.S., Lee W.J. (2013). Vitamin C Is an Essential Factor on the Anti-viral Immune Responses through the Production of Interferon-alpha/beta at the Initial Stage of Influenza A Virus (H3N2) Infection. Immune Netw..

[9] Hemila H., de Man A. (2020). Vitamin C and COVID-19. Front. Med..

[10] Zhang J., Rao X., Li Y., Zhu Y., Liu F., Guo G., Luo G., Meng Z., De Backer D., Xiang H. (2021). Pilot trial of high-dose vitamin C in critically ill COVID-19 patients. Ann. Intensive Care.

[11] Carr A.C., Maggini S. (2017). Vitamin C and Immune Function. Nutrients.

[12] Holtzman M.J., Byers D.E., Alexander-Brett J., Wang X. (2014). The role of airway epithelial cells and innate immune cells in chronic respiratory disease. Nat. Rev. Immunol..

[13] Agrawal S., Srivastava R., Rahmatpanah F., Madiraju C., BenMohamed L., Agrawal A. (2017). Airway Epithelial Cells Enhance the Immunogenicity of Human Myeloid Dendritic Cells under Steady State. Clin. Exp. Immunol..

[14] Rahmatpanah F., Agrawal S., Jaiswal N., Nguyen H.M., McClelland M., Agrawal A. (2019). Airway epithelial cells prime plasmacytoid dendritic cells to respond to pathogens via secretion of growth factors. Mucosal. Immunol..

[15] Subramanian V.S., Sabui S., Moradi H., Marchant J.S., Said H.M. (2018). Inhibition of intestinal ascorbic acid uptake by lipopolysaccharide is mediated via transcriptional mechanisms. Biochim. Biophys. Acta. Biomembr..

[16] Subramanian V.S., Sabui S., Subramenium G.A., Marchant J.S., Said H.M. (2018). Tumor necrosis factor alpha reduces intestinal vitamin C uptake: A role for NF-κB-mediated signaling. Am. J. Physiol. Gastrointest. Liver Physiol..

[17] Alldredge J., Randall L., De Robles G., Agrawal A., Mercola D., Liu M., Randhawa P., Edwards R., McClelland M., Rahmatpanah F. (2020). Transcriptome Analysis of Ovarian and Uterine Clear Cell Malignancies. Front. Oncol..

[18] Samarajiwa S.A., Forster S., Auchettl K., Hertzog P.J. (2009). INTERFEROME: The database of interferon regulated genes. Nucleic Acids Res..

[19] Rusinova I., Forster S., Yu S., Kannan A., Masse M., Cumming H., Chapman R., Hertzog P.J. (2013). Interferome v2.0: An updated database of annotated interferon-regulated genes. Nucleic Acids Res..

[20] Maeda N., Hagihara H., Nakata Y., Hiller S., Wilder J., Reddick R. (2000). Aortic wall damage in mice unable to synthesize ascorbic acid. Proc. Natl. Acad. Sci. USA.

[21] Larsson N., Rankin G.D., Bicer E.M., Roos-Engstrand E., Pourazar J., Blomberg A., Mudway I.S., Behndig A.F. (2015). Identification of vitamin C transporters in the human airways: A cross-sectional in vivo study. BMJ Open.

[22] Caput D., Beutler B., Hartog K., Thayer R., Brown-Shimer S., Cerami A. (1986). Identification of a common nucleotide sequence in the 3′-untranslated region of mRNA molecules specifying inflammatory mediators. Proc. Natl. Acad. Sci. USA.

[23] de Toeuf B., Soin R., Nazih A., Dragojevic M., Jurenas D., Delacourt N., Vo Ngoc L., Garcia-Pino A., Kruys V., Gueydan C. (2018). ARE-mediated decay controls gene expression and cellular metabolism upon oxygen variations. Sci. Rep..

[24] Takayama S., Reed J.C. (2001). Molecular chaperone targeting and regulation by BAG family proteins. Nat. Cell Biol..

[25] Arndt V., Daniel C., Nastainczyk W., Alberti S., Hohfeld J. (2005). BAG-2 acts as an inhibitor of the chaperone-associated ubiquitin ligase CHIP. Mol. Biol. Cell.

[26] Fei X., Ziqian Y., Bingwu Y., Min L., Xinmiao X., Zhen M., Lirong G., Song W. (2021). Aldosterone alleviates lipopolysaccharide-induced acute lung injury by regulating epithelial sodium channel through PI3K/Akt/SGK1 signaling pathway. Mol. Cell. Probes.

[27] Yang E., Li M.M.H. (2020). All About the RNA: Interferon-Stimulated Genes That Interfere with Viral RNA Processes. Front. Immunol..

[28] Pozharskaya V., Torres-Gonzalez E., Rojas M., Gal A., Amin M., Dollard S., Roman J., Stecenko A.A., Mora A.L. (2009). Twist: A regulator of epithelial-mesenchymal transition in lung fibrosis. PLoS ONE.

[29] Rehwinkel J., Gack M.U. (2020). RIG-I-like receptors: Their regulation and roles in RNA sensing. Nat. Rev. Immunol..

[30] Hemila H. (2017). Vitamin C and Infections. Nutrients.

[31] Valero N., Mosquera J., Alcocer S., Bonilla E., Salazar J., Alvarez-Mon M. (2015). Melatonin, minocycline and ascorbic acid reduce oxidative stress and viral titers and increase survival rate in experimental Venezuelan equine encephalitis. Brain Res..

[32] Cai Y., Li Y.F., Tang L.P., Tsoi B., Chen M., Chen H., Chen X.M., Tan R.R., Kurihara H., He R.R. (2015). A new mechanism of vitamin C effects on A/FM/1/47(H1N1) virus-induced pneumonia in restraint-stressed mice. Biomed. Res. Int..

[33] Li W., Maeda N., Beck M.A. (2006). Vitamin C deficiency increases the lung pathology of influenza virus-infected gulo^-/-^ mice. J. Nutr..

[34] Fischer H., Schwarzer C., Illek B. (2004). Vitamin C controls the cystic fibrosis transmembrane conductance regulator chloride channel. Proc. Natl. Acad. Sci. USA.

[35] Traber M.G., Stevens J.F. (2011). Vitamins C and E: Beneficial effects from a mechanistic perspective. Free Radic. Biol. Med..

[36] Uchide N., Toyoda H. (2011). Antioxidant therapy as a potential approach to severe influenza-associated complications. Molecules.

[37] Castro S.M., Guerrero-Plata A., Suarez-Real G., Adegboyega P.A., Colasurdo G.N., Khan A.M., Garofalo R.P., Casola A. (2006). Antioxidant treatment ameliorates respiratory syncytial virus-induced disease and lung inflammation. Am. J. Respir. Crit. Care Med..

[38] Liu Y., Wang M., Cheng A., Yang Q., Wu Y., Jia R., Liu M., Zhu D., Chen S., Zhang S. (2020). The role of host eIF2alpha in viral infection. Virol. J..

[39] Choi Y., Bowman J.W., Jung J.U. (2018). Autophagy during viral infection—A double-edged sword. Nat. Rev. Microbiol..

[40] Schneider W.M., Chevillotte M.D., Rice C.M. (2014). Interferon-stimulated genes: A complex web of host defenses. Annu. Rev. Immunol..

[41] Bailey C.C., Zhong G., Huang I.C., Farzan M. (2014). IFITM-Family Proteins: The Cell’s First Line of Antiviral Defense. Annu. Rev. Virol..

[42] Aumiller V., Balsara N., Wilhelm J., Gunther A., Konigshoff M. (2013). WNT/beta-catenin signaling induces IL-1beta expression by alveolar epithelial cells in pulmonary fibrosis. Am. J. Respir. Cell Mol. Biol..

[43] Hussain M., Xu C., Lu M., Wu X., Tang L., Wu X. (2017). Wnt/beta-catenin signaling links embryonic lung development and asthmatic airway remodeling. Biochim. Biophys. Acta Mol. Basis Dis..

[44] Lu C., Huang T., Chen W., Lu H. (2015). GnRH participates in the self-renewal of A549-derived lung cancer stem-like cells through upregulation of the JNK signaling pathway. Oncol. Rep..

[45] Patterson T., Isales C.M., Fulzele S. (2021). Low level of Vitamin C and dysregulation of Vitamin C transporter might be involved in the severity of COVID-19 Infection. Aging Dis..

[46] Hemilä H., Chalker E. (2019). Vitamin C Can Shorten the Length of Stay in the ICU: A Meta-Analysis. Nutrients.

[47] Marik P.E., Khangoora V., Rivera R., Hooper M.H., Catravas J. (2017). Hydrocortisone, Vitamin C, and Thiamine for the Treatment of Severe Sepsis and Septic Shock: A Retrospective Before-After Study. Chest.

[48] Patel V., Dial K., Wu J., Gauthier A.G., Wu W., Lin M., Espey M.G., Thomas D.D., Ashby C.R., Mantell L.L. (2020). Dietary Antioxidants Significantly Attenuate Hyperoxia-Induced Acute Inflammatory Lung Injury by Enhancing Macrophage Function via Reducing the Accumulation of Airway HMGB1. Int. J. Mol. Sci..

[49] Thomas S., Patel D., Bittel B., Wolski K., Wang Q., Kumar A., Il’Giovine Z.J., Mehra R., McWilliams C., Nissen S.E. (2021). Effect of High-Dose Zinc and Ascorbic Acid Supplementation vs. Usual Care on Symptom Length and Reduction Among Ambulatory Patients With SARS-CoV-2 Infection: The COVID A to Z Randomized Clinical Trial. JAMA Netw. Open.

